# Cardiovascular Disease, Single Nucleotide Polymorphisms; and the Renin Angiotensin System: Is There a MicroRNA Connection?

**DOI:** 10.4061/2010/281692

**Published:** 2010-08-04

**Authors:** Terry S. Elton, Sarah E. Sansom, Mickey M. Martin

**Affiliations:** ^1^Davis Heart and Lung Research Institute, The Ohio State University, DHLRI 515, Columbus, OH 43210, USA; ^2^Division of Pharmacology, College of Pharmacy, The Ohio State University, OH 43210, USA; ^3^Department of Medicine, College of Medicine, Division of Cardiology, The Ohio State University, OH 43210, USA

## Abstract

Essential hypertension is a complex disorder, caused by the interplay between many genetic variants, gene-gene interactions, and environmental factors. Given that the renin-angiotensin system (RAS) plays an important role in blood pressure (BP) control, cardiovascular regulation, and cardiovascular remodeling, special attention has been devoted to the investigation of single-nucleotide polymorphisms (SNP) harbored in RAS genes that may be associated with hypertension and cardiovascular disease. MicroRNAs (miRNAs) are a family of small, ∼21-nucleotide long, and nonprotein-coding RNAs that recognize target mRNAs through partial complementary elements in the 3′-untranslated region (3′-UTR) of mRNAs and inhibit gene expression by targeting mRNAs for translational repression or destabilization. Since miRNA SNPs (miRSNPs) can create, destroy, or modify miRNA binding sites, this review focuses on the hypothesis that transcribed target SNPs harbored in RAS mRNAs, that alter miRNA gene regulation and consequently protein expression, may contribute to cardiovascular disease susceptibility.

## 1. Introduction

Identifying the genes and mutations that contribute to disease is a central aim in human genetics. Single nucleotide polymorphisms (SNPs) are mutations that occur at genome positions at which there are two distinct nucleotide residues (alleles) that each appear in a significant portion (i.e., a minor allele frequency greater than 1%) of the human population [1]. There are some estimated 14 million SNPs [2] in the human genome that occur at a frequency of approximately one in 1,200–1,500 bp [3]. SNPs can affect protein function by changing the amino acid sequences (nonsynonymous SNP) or by perturbing their regulation (e.g., affecting promoter activity [4], splicing process [5], and DNA and pre-mRNA conformation). When SNPs occur in 3′-UTRs, they may interfere with mRNA stability and translation by altering polyadenylation and protein/mRNA regulatory interactions. Recently, a new layer of posttranscriptional miRNA-mediated gene regulation has been discovered and shown to control the expression levels of a large proportion of genes (reviewed in [6]). Importantly, SNPs in microRNA (miRNA) target sites (miRSNPs) represent a specific class of regulatory polymorphisms in the 3′-UTR that may lead to the dysregulation of posttranscriptional gene expression. Thus, for miRNAs acting by this mechanism, the miRSNPs may lead to heritable variations in gene expression. Given that the renin angiotensin system (RAS) is intricately involved in the pathogenesis of cardiovascular disease [7–12], we review and discuss the presently available evidence for miRSNPs-mediated RAS gene regulation and its importance for phenotypic variation and disease.

## 2. Current View of the Renin Angiotensin System

The RAS plays a critical role in regulating the physiological processes of the cardiovascular system [reviewed in [7–14]]. The primary effector molecule of this system, angiotensin II (Ang II), has emerged as a critical hormone that affects the function of virtually all organs, including heart, kidney, vasculature, and brain, and it has both beneficial and pathological effects [7–14]. Acute stimulation with Ang II regulates salt/water homeostasis and vasoconstriction, modulating blood pressure, while chronic stimulation promotes hyperplasia and hypertrophy of vascular smooth muscle cells (VSMCs). In addition, long-term exposure to Ang II also plays a pathophysiological role in cardiac hypertrophy and remodeling, myocardial infarction, hypertension, atherosclerosis, in-stent restenosis, reduced fibrinolysis, and renal fibrosis [7–14].

**Figure 1:**
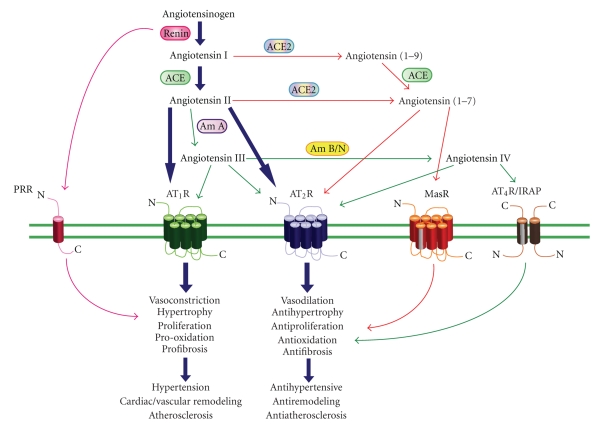
Summary of the RAS incorporating the Ang peptide family and physiological effects mediated via ATR subtypes. Under the classical RAS schema, Ang II is produced, via renin and ACE, to act with equal affinity on two ATR subtypes, AT_1_R and AT_2_R (large arrows). However, it is now appreciated that a number of breakdown products of Ang II, namely, Ang (1–7), Ang III, and Ang IV exert their own unique effects that are distinct (and often opposite) to those of Ang II. Such effects are often mediated via newly recognized receptors such as MasR for Ang (1–7) and AT_4_R (also known as IRAP) for Ang IV, or additionally via AT_2_R stimulation. ACE2 is also a new pathway for the formation of Ang (1–7). Newly identified Ang receptor binding proteins associated with different ATR subtypes may also modify ATR activation. Thus, overstimulation of AT_1_R (and PRR) by Ang II, which can contribute to a plethora of cardiovascular disease processes, may be counter-regulated by a number of non-AT_1_R mechanisms. Most notably, AT_2_R stimulation usually causes opposing effects to AT_1_R, as indicated. It is also likely that the MasR exerts a similar counter-regulatory role whereas the evidence is more preliminary and speculative for AT_4_R/IRAP. In terms of mediators, Ang II itself stimulates AT_2_R whereas the shorter Ang peptides stimulate their cognate receptors and possibly also AT_2_R.

Ang II, an octapeptide hormone, is produced systemically via the classical RAS and locally via the tissue RAS [7–14]. In the classical RAS, circulating renal-derived renin cleaves hepatic-derived angiotensinogen to form the decapeptide angiotensin I (Ang I), which is converted by angiotensin-converting enzyme (ACE) in the lungs to the biologically active Ang II (Figure 1). Alternatively, a recently identified carboxypeptidase, ACE2, cleaves one amino acid from either Ang I or Ang II [15–18], decreasing Ang II levels and increasing the metabolite Ang 1–7, which has vasodilator properties. Thus, the balance between ACE and ACE2 is an important factor controlling Ang II levels [15–18]. Ang II is also further degraded by aminopeptidases to Ang III (Ang 2–8) and Ang IV (Ang 3–8) (Figure 1) [7]. Although the RAS was originally regarded as a circulating system, many of its components are localized in tissues, including the heart, brain, blood vessels, adrenal, kidney, liver and reproductive organs, indicating the existence of local tissue RASs [19]. In addition to ACE-dependent pathways of Ang II formation, non-ACE pathways have also been described. Chymotrypsin-like serine protease (chymase) may represent an important mechanism for conversion of Ang I to Ang II in the human heart, kidney, and vasculature and may be particularly important in pathological conditions such as coronary heart disease [20].

The biological responses to Ang II are mediated by its interaction with two distinct high-affinity G protein-coupled receptors (GPCRs) designated AT_1_R and AT_2_R (Figure 1) [7]. Both AT_1_R and AT_2_R possess similar affinity for Ang II [21]; however, pharmacologically, these receptors can be distinguished according to inhibition by specific antagonists. For example, AT_1_R are selectively antagonized by biphenylimidazoles such as losartan (angiotensin receptor blockers, ARB) [21] whereas tetrahydroimidazopyridines such as PD123319 specifically inhibit AT_2_R [21, 22]. Interestingly, all of the classical actions of Ang II, including vasoconstriction, effects on fluid and electrolyte homeostasis, and influences on cellular growth and differentiation, have been shown to be due to stimulation of AT_1_R located on the plasma membrane of cells [7–12]. Additionally, the majority of the pathophysiological effects (i.e., cardiac hypertrophy and remodeling, myocardial infarction, hypertension, etc.) of Ang II are also mediated via the AT_1_R [7–12]. In contrast, it is thought that the AT_2_R counter-regulates AT_1_R function (reviewed in [13, 14]). It is also speculated that during cardiovascular disease, AT_2_R upregulation and activation by Ang II, or angiotensin peptide fragments (i.e., Ang III, Ang IV, and/or Ang 1–7) may limit AT_1_R-mediated overactivity and cardiovascular pathologies [13, 14]. 

Although the AT_1_R and AT_2_R have been intensively investigated it is now clear that angiotensin fragments can bind to and activate other receptor subtypes. For example, Ang 1–7 acts on the Mas GPCR (MasR) and has vasodilatory and antiproliferative effects. This arm of the RAS is also thought to counterbalance the effects of Ang II acting on the AT_1_R (Figure 1) (reviewed in [18]). Additionally, Ang IV can bind to the angiotensin II type 4 receptor (AT_4_R) or the membrane-bound, insulin-regulated aminopeptidase (IRAP) (Figure 1) and mediate the enhancement of cognitive function, modulate blood flow, increase natriuresis, inhibit cardiomyocyte hypertrophy, and improve endothelial function in animal models of atherosclerosis [23, 24].

Finally, recent studies now suggest that renin, the aspartyl protease that cleaves angiotensinogen into Ang I, and prorenin, its proenzyme inactive form, can bind to what is now designated as the (pro)renin receptor (PRR) (reviewed in [25–27]). Interestingly, the binding of renin/prorenin to PRR has been shown to have two major consequences. First, the binding of renin to its receptor increases angiotensinogen conversion to Ang I by five-fold, and prorenin, which is virtually inactive in solution, also displays enzymatic activity following receptor binding [25–27]. Second, receptor-bound renin/prorenin activates the MAP kinases ERK1/2 and p38 pathways, which in turn, leads to the upregulation of profibrotic and cyclooxygenase-2 genes independent of Ang II generation [25–27]. Therefore, the activation and potentiation of renin/prorenin enzymatic activity, together with specific PRR-mediated signaling, could have striking effects on cardiovascular regulation. Taken together, these studies suggest that RAS is unexpectedly complex and multilayered. New components and functions of the RAS are still being unraveled and the physiological significance, and ultimately the clinical relevance, of these factors remain largely undefined.

## 3. Overview of miRNA Biology

MicroRNAs (miRNAs) are endogenous, short (20–23 nucleotide), and single-stranded nonprotein-coding RNA molecules that regulate gene expression (reviewed in [28]). These molecules act by binding to their target mRNAs, preferentially to the 3′-UTR, using a partial base-pairing mechanism. In order for a miRNA to give rise to functional consequences, the 7-8 nucleotides (nt) at the most 5′ end must have exact complementarity to the target mRNA, generally referred to as the “seed” region [29]. The current model for inhibition of expression by a miRNA suggests that a miRNA either inhibits translation or induces degradation of its target mRNA, depending upon the overall degree of complementarity of the binding site, number of binding sites, and the accessibility of those binding sites [30–32]. 

In mammals, computational predictions indicate that miRNAs may regulate 60% of all human protein coding genes [33], and have been increasingly implicated in the control of various biological processes, including cell differentiation, cell proliferation, development and apoptosis, and many pathological processes such as cancer, Alzheimer's disease, and cardiovascular disease [34–36]. There are estimated to be >1,000 miRNAs encoded by the human genome [37, 38], each of which can act on multiple target mRNAs. Conversely, individual mRNAs are commonly targeted by multiple miRNAs, which results in a combinatorial repression of gene expression more robust than the suppression that results from a single miRNA [39, 40]. Although miRNAs are known to mediate posttranscriptional gene silencing in the cytoplasm, recent evidence indicates that at least some fraction of mammalian miRNAs may also activate or inhibit gene expression at the transcriptional level [41, 42]. Taken together, these miRNA phenomena allow for enormous combinatorial complexity and regulatory potential.

## 4. miRNA Biogenesis

**Figure 2:**
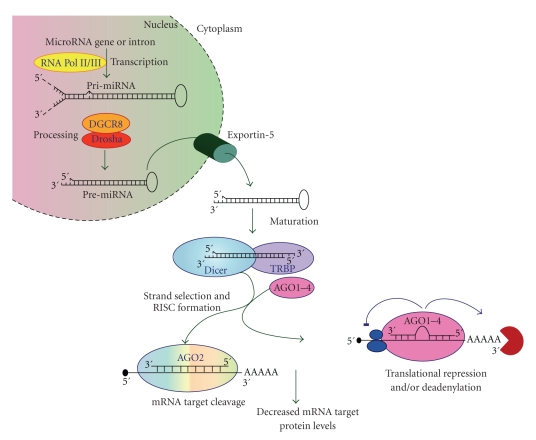
Schematic representation outlining miRNA biogenesis including transcription, maturation, and miRNA/mRNA targeting. This diagram also outlines two potential mechanisms for miRNA/mRNA silencing. The specific details discussing these processes are included in the text.

Mature miRNAs are processed from primary miRNA transcripts (pri-miRNAs), which are either transcribed from independent miRNA genes or are portions of introns of protein-coding RNA polymerase II transcripts (Figure 2) [43–45]. miRNAs tend to cluster throughout the genome and many of these clusters are likely transcribed as polycistrons [46–48]. Although little is known regarding the regulation of miRNA transcription, it is recognized that miRNA expression is usually regulated by established transcriptional mechanisms. Interestingly, however, it has been shown that each miRNA located within the same genomic cluster may be transcribed and regulated independently [49].

During the transcriptional process, pri-miRNAs fold into hairpin structures containing imperfectly base-paired stems and are endonucleolytically cleaved by the nuclear microprocessor complex formed by the RNase III type endonuclease Drosha and the DiGeorge critical region 8 (DGCR8) protein [50]. The Drosha/DGCR8 complex processes pri-miRNAs into ~70-nucleotide hairpins known as pre-miRNAs (Figure 2) [28, 51]. In animals, pre-miRNAs are exported from the nucleus to the cytoplasm via Exportin-5, where they are cleaved by Dicer complexed with the TAR RNA binding protein (TRBP), to yield ~20-bp miRNA duplexes. In principle, the miRNA duplex could give rise to two different mature miRNAs. However, in a manner similar to siRNA duplexes, only one strand is usually incorporated into miRNA-induced silencing complexes (miRISCs) and guides the complex to target mRNAs; the other strand is degraded (the complementary miRNA* strand) [52]. This functional asymmetry depends on the thermodynamic stability of the base pairs at the two ends of the duplex, with the miRNA strand, which has the least stable base pair at its 5′ end in the duplex, being loaded into the miRISC [53]. Still, recent data suggest that both arms of the pre-miRNA hairpin can give rise to mature miRNAs [28, 45, 51].

## 5. miRNA/mRNA Silencing

Although the details are not well understood, pre-miRNA processing by Dicer is coupled with the assembly of miRNAs into ribonucleoprotein complexes called micro-RNPs (miRNPs) or miRISCs [28, 51, 54]. One of the key components of miRNPs is the Argonaute (AGO) protein family, AGO1 to AGO4, and while all four AGO proteins function in miRNA repression only AGO2 functions in mRNA target cleavage (Figure 2) [54, 55]. Once the miRNA is processed, the mature miRNA acts as an adaptor for miRISC to specifically recognize and regulate particular mRNAs. It is currently thought that the miRNA-loaded RISC is targeted to a given mRNA by a mechanism where the miRISC binds many sites nonspecifically until the correct target site is found [56].

With few exceptions, miRNA binding sites in animal mRNAs are present in the 3′-UTR and mature miRNAs base pair with their target mRNAs imperfectly, following a set of rules that have been identified by experimental and bioinformatics analyses [29, 57–60]. First, miRNA/mRNA target recognition involves Watson-Crick base pairing that must be perfect and contiguous at the 5′-end of the miRNA from nucleotides 2 to 8. This section represents the “seed” region and nucleates the miRNA-mRNA association. Second, G:U wobble pairing in the seed sequence is highly detrimental to miRNA function despite its favorable contribution to RNA:RNA duplexes. Third, an A residue across position 1 of the miRNA, and an A or U across position 9, improve the site efficiency, although they do not need to base pair with miRNA nucleotides. Fourth, it has been established that miRNAs that have suboptimal 5′ Watson-Crick base pairing need substantial complementarity to the miRNA 3′ half to stabilize the interaction and to be functional. Finally, the context of the miRNA's binding sites harbored in the 3′-UTR of target mRNAs, also influence the functional importance of these sites [61]. For example, miRNA site efficacy can be improved if the site is positioned at least 15 nt downstream from the stop codon, away from the center of long 3′-UTRs, and near AU-rich nucleotide regions. These factors can make the 3′-UTR regions less structured and hence more accessible to miRNP recognition.

When an endogenous miRISC programmed with miRNA binds to a recognition site that is perfectly complementary, this target mRNA will be cleaved by the miRISC (Figure 2) [56, 62–65]. In contrast, miRISCs that are imperfectly matched with target mRNAs can repress translation initiation at either the cap-recognition stage [66–70] or the 60S subunit joining stage [71]. Alternatively, binding of miRISCs can induce deadenylation and decay of target mRNAs [62].

## 6. Computational Algorithms to Predict miRNA/mRNA Targets

Computational miRNA/mRNA target programs remain the only source for rapid prediction of miRNA recognition sites harbored within the 3′-UTR of target mRNAs. Therefore, the development of reliable computational target prediction programs is critical in advancing our understanding of miRNA function. Given that miRNA functionality usually requires seed sequence complementarity [28, 61] the main prediction feature used in most of these programs is the sequence alignment of the miRNA seed to the 3′-UTR of candidate target genes. Additionally, many current algorithms also utilize conservation of miRNA/mRNA target sites across species as an important parameter for the identification of bona fide targets; notably however, the conservation of a miRNA binding site harbored in a given mRNA target is not a requirement for a functional miRNA.

A recent review article [72] comparing eight of the most commonly used algorithms for miRNA target prediction for the human and mouse genome programs demonstrated that the four top algorithms, DIANA-microT 3.0 (http://microrna.gr/microT) [73], TargetScan 5.0 (http://www.targetscan.org/) [74], Pictar (http://pictar.org/) [75], and ElMMo (from http://www.mirz.unibas.ch) [76] all have a precision of ~50% with a sensitivity that ranges from 6% to 12%. Of the top four performing programs, it is important to note that TargetScan is the most up-to-date regarding the number of miRNAs and genes used and Pictar is least updated [72]. Most investigators assume that mRNA targets predicted by more than one algorithm are more accurate than other targets thus leading to higher prediction precision. However, Alexiou et al. [72] demonstrated that many of the algorithm combinations performed worse than the prediction of a single algorithm. These investigators reason that the better specificity of a combination is achieved by a higher price for the sensitivity. Taking this into account, our laboratory has analyzed all of the classical and nonclassical RAS components for putative miRNA binding sites by the TargetScan algorithm (Table 1). Importantly, this analysis suggests that miRNAs may play a major role in regulating the expression of RAS proteins.

Although many programs are available online for the prediction of individual mRNA targets of miRNAs (see above), the identification of authentic mRNA targets remains problematic. Mammalian miRNAs bind to the mRNA with imperfect complementarity thus how binding sites are recognized is only partially understood. Therefore, some bioinformatically predicted targets turn out to be false and others are entirely overlooked. Experimental validation of targets is therefore an important step in defining the functions of individual miRNAs (for review, see [77]).

## 7. Experimentally Validated miRNA/RAS Targets

Although classical and nonclassical RAS components harbor putative miRNA binding sites, very few of these sites have been experimentally validated. Our laboratory has demonstrated that miR-155 specifically interacted with the algorithm-predicted binding site harbored in the 3′-UTR of the human AT_1_R (hAT_1_R) mRNA (Table 1) [78, 79]. Additionally, miR-155 gain-of-function experiments (i.e., cells were transfected with partially double-stranded RNAs that mimic the Dicer cleavage product and are subsequently processed into their respective mature miRNAs) inhibited the expression of the hAT_1_R and also attenuated Ang II-induced signaling via the hAT_1_R in fibroblasts and vascular smooth muscle cells (VSMCs) [78, 79]. These results also demonstrated that transfection with miR-155 did not significantly decrease hAT_1_R steady state mRNA levels, suggesting that miR-155 can decrease hAT_1_R expression by inhibiting translation of the mRNA, rather than targeting it for degradation. In contrast, loss-of-function experiments (i.e., cells were transfected with miRNA inhibitors; antisense single-stranded chemically-enhanced oligonucleotides, ASO) demonstrated that transfection of anti-miR-155 not only increased hAT_1_R expression but also enhanced Ang II-induced signaling via the hAT_1_R, indicating that miR-155 plays a physiological role in regulating the expression of hAT_1_Rs in human fibroblasts and VSMCs [78, 79]. Recently, our laboratory also demonstrated that hAT_1_R expression can be regulated by miR-802 [80]. 

In support of the miR-155/hAT_1_R studies described above, trisomy 21 (Ts21) mediated overexpression of miR-155 (the bic/miR-155 gene is located on human chromosome 21 and is triplicated in Down syndrome [DS] individuals) [81, 82] resulted in the attenuation of hAT_1_R protein levels in fibroblasts isolated from one monozygotic twin with DS when compared to fibroblasts isolated from the unaffected euploid twin [83]. Interestingly, individuals with DS have significantly lower systolic and diastolic blood pressures [84–87], a reduced risk of vascular anomalies [88], and a low prevalence of coronary artery disease [89–93] when compared with the general population. Given that the over-expression of miR-155 in Ts21 results in attenuated hAT_1_R protein levels, we speculate that this may be one mechanism which contributes to the lack of cardiovascular disease observed in individuals with DS [84–87]. 

Finally, Boettger et al. [94] demonstrated by genomic, proteomic, and transcriptional analyses that mouse ACE mRNA is a miR-145 target. The miR-143/-145 gene cluster includes miR-143 and miR-145, which lie within a 1.7-kb highly conserved region of mouse chromosome 18 [94]. miRNA microarray hybridization experiments revealed that miR-143 and miR-145 are enriched in murine vascular smooth muscle cells (VSMCs) [94, 95]. In the mouse embryo, miR-143/-145 expression is restricted to heart, vascular, and visceral SMCs [94, 95]. During late fetal and postnatal development, miR-143/-145 expression is downregulated in the heart but persists in vascular and visceral SMCs. Homozygous miR-143/-145 knockout mice were viable but exhibited thinning of the arterial tunica media (muscular layer), with a reduction in the number of contractile VSMCs and a concomitant increase in the number of proliferative VSMCs [94]. Physiological characterization of miR-143/-145 deficient mice and arterial segments from these animals revealed defects in Ang II-induced VSMC contractility and homeostatic control of blood pressure. Consistent with this observation, pharmacological inhibition of ACE or the AT_1_R partially reversed vascular dysfunction and normalized gene expression in the mutant mice [94]. Since miR-145 regulates the expression of ACE, when the miR-143/-145 gene cluster is mutated, the levels of membrane-bound ACE in VSMCs increase, causing the chronic stimulation of VSMCs by Ang II, which in turn results in desensitization and “angiotensin resistance” of VSMCs. Given that miR-143/145 mutant mice developed neointimal lesions in the absence of hyperlidemia, lipid depositions, and foam cells also highlights the potential role of VSMCs in the pathogenetic process leading to atherosclerosis. The enhancement of Ang II signaling due to the increased expression of ACE would certainly contribute to this process, since increased levels of Ang II have been shown to promote atherosclerotic lesions in apoE-deficient mice [96].

Although the hAT_1_R and mouse ACE are experimentally validated targets of miRNAs, it is important to note that none of these recognition sites are conserved across species (Table 1). Therefore, although miR-155 and -802 repress hAT_1_R expression in humans, these miRNAs will not lead to the repression of AT_1_R levels in mice or rats. Likewise, miR-145 will repress ACE expression in mice but will not regulate ACE levels in humans.

## 8. miRSNPs

Since a large number of miRNA binding sites are harbored in the 3′-UTRs of RAS component mRNAs (Table 1), there is a high probability that SNPs will occur within miRNA target sites. By definition, miRSNPs have the potential to create, destroy, or modify the efficiency of miRNA binding, if the SNP occurs in the seed region (i.e., the region of base-pairing between nucleotides 2 and 8 of the miRNA and complementary nucleotides in the target mRNA) [97–99]. Based on this definition, there are two mechanisms by which miRSNPs can be functionally important: as a gain- or as a loss-of-function variation. A gain-of-function effect would result if the SNP enhances the targeting of the miRNA or creates a new target site in the 3′-UTR of the mRNA. In this scenario, protein expression of the target mRNA would be attenuated. In contrast, a loss-of-function effect would result when the SNP decreases or abolishes the interaction of the miRNA with its mRNA target, thus resulting in an augmentation of protein expression. In support of this conclusion, miRSNPs have been implicated in Tourette syndrome [100], papillary thyroid cancer [101], muscularity in sheep [102], hereditary spastic paraplegia type 31 [103], methotrexate resistance [104], breast cancer [105], and tumor susceptibility [106]. These examples include both gain- and loss-of-function miRSNPs.

## 9. The Human AT_1_R Gene and miRSNPs

**Figure 3:**
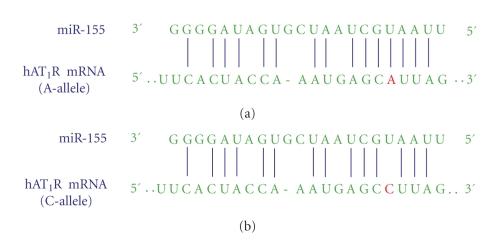
The human AT_1_R +1166 A/C SNP occurs in the miR-155-binding site. (a) Complementarity between miR-155 and the hAT_1_R 3′-UTR site targeted (70–90 bp downstream from the human AT_1_R stop codon). The +1166 A/C SNP corresponds to the nucleotide 86 bp downstream from the human AT_1_R stop codon (shown in red print). The binding of miR-155 to the hAT_1_R 3′-UTR target site fulfills the requirement of a 7-bp seed sequence of complementarity at the miRNA 5′ end when the +1166 A-allele is expressed. (b) Complementarity between miR-155 and the human AT_1_R 3′-UTR harboring the +1166 C-allele. If the +1166 C-allele is expressed, the seed sequence requirement would not be met and, as a consequence, it would be expected that human AT_1_R expression would be elevated [79].

The hAT_1_R gene has been found to be highly polymorphic [107]. In particular, a SNP has been described in which there is an A/C transversion at position +1166 (i.e., 1166 base-pairs downstream from the start codon, dsSNP# rs5186) located in the 3′-UTR of the hAT_1_R gene. The increased frequency of the +1166 C-allele has been associated with hypertension [108–115], cardiac hypertrophy [116–118], aortic stiffness [119–121], myocardial infarction [122], heart failure [123–125], abdominal aortic aneurysms [126, 127], and increased oxidative stress levels in human heart failure [128]. However, the physiological relevance of this polymorphism is uncertain because of its location within the noncoding region of the hAT_1_R gene. Since our laboratory has previously demonstrated that miR-155 interacted with a specific *cis*-response element localized in the hAT_1_R 3′-UTR [78], we investigated whether or not there was a correlation between the +1166 A/C SNP and the miR-155-binding site [79, 83]. Importantly, computer alignment revealed that the +1166 A/C SNP occurs within the *cis*-response element at the site where miR-155 was shown to interact (Figure 3). The interaction between miR-155 and the hAT_1_R 3′-UTR harboring the A-allele fulfills the seed sequence rules [29] since there is a 7-bp region of complementarity between the 5′ end of miR-155 and the hAT_1_R mRNA target site (Figure 3(a)). In contrast, if a hAT_1_R mRNA that harbors the +1166 C-allele is expressed, the complementary seed site is interrupted (Figure 3(b)), and the thermodynamics of the miRNA:mRNA duplex would be significantly altered (i.e., a decrease in free energy) [79]. Therefore, the presence of the +1166 C-allele miRSNP would decrease the ability of miR-155 to interact with the *cis*-regulatory site located in the hAT_1_R 3′-UTR. As a consequence, it would be expected that aberrantly high levels of the hAT_1_R would be synthesized. In support of this hypothesis we demonstrated that when the hAT_1_R *cis*-response element harboring the C-allele was present in luciferase mRNAs, the ability of miR-155 to inhibit luciferase activity was significantly attenuated [79]. When identical experiments were performed utilizing mutant miR-155, which restored perfect Watson-Crick complementarity, luciferase activity was decreased to levels that were comparable with experiments utilizing the hAT_1_R *cis*-response element harboring the A-allele and miR-155 [79]. To further demonstrate that the presence of the +1166 C-allele can influence hAT_1_R density, expression constructs that produced hAT_1_R mRNAs containing either the A- or C-allele were cotransfected with miR-155 or mut-miR-155. These experiments again demonstrated that when seed sequence complementarity was not fulfilled, regardless of whether miR-155 or mutant miR-155 was utilized, hAT_1_R levels were always higher than the levels obtained when perfect complementarity was present between the miRNA and the hAT_1_R *cis*-response element [79]. Taken together, these studies provide the first feasible biochemical mechanism by which the +1166 A/C polymorphism (i.e., miRSNP) can lead to increased hAT_1_R densities and possibly cardiovascular disease.

## 10. miRSNPS and RAS Component Genes

To begin to investigate whether other RAS components harbored SNPs in their 3′-UTRs which created miRSNPs, the SNP Geneview Report (http://www.ncbi.nlm.nih.gov/nucleotide/) for each gene was surveyed and all of the identified SNPs were analyzed utilizing the Patrocles algorithm (http://www.patrocles.org/) [99]. Specifically the “Patrocles Finder” was utilized since it allows one to compare two sequences and subsequently determines the miRNA binding sites that are different between the two sequences. Thus, a region of the transcribed 3′-UTR which harbored the common allele was compared with the same region containing the mutated allele (select motif length: human 7 mers) and the generation of putative miRSNPs was determined (Table 2). Importantly, this analysis demonstrated that the 3′-UTRs of the RAS components harbor a number of miRSNPs some of which alter or destroy legitimate miRNA binding sites and others that create novel, illegitimate target sites. Hypothetically, if a loss-of-function miRSNP occurred in the AGTR1, ACE, AGT, REN, or ATP6AP2 gene, an increased incidence of hypertension, cardiac/vascular remodeling, and atherosclerosis would be observed (Table 3). In contrast, if a loss-of-function miRSNP occurred in the AGTR2, ACE2, MAS1, or LNPEP gene, a decreased incidence of hypertension, cardiac/vascular remodeling, and atherosclerosis would be expected (Table 3).

## 11. Conclusion

miRNAs have been increasingly implicated in the control of various biological processes, including cell differentiation, cell proliferation, development and apoptosis, and many pathological processes such as cancer, Alzheimer's disease, and cardiovascular disease [34–36]. Importantly, several studies which have now demonstrated that polymorphisms at the miRNA target site in the 3′-UTRs of transcribed mRNAs can have detrimental effects given that miRSNPs can lead to the modulation of gene expression [79, 83, 100–106]. In support of this hypothesis, a recent study suggested that differences in SNP allele frequency among ethnic groups account for differences in gene expression [129]. Therefore, we speculate that miRSNPs can modulate RAS phenotypic gene expression diversities, at least in part, through alteration of miRNA target binding capability, ultimately leading to differences in the susceptibility to complex genetic disorders, such as cardiovascular disease.

## Figures and Tables

**Table 1:** TargetScan* algorithm-predicted RAS putative miRNA/mRNA target sites.

RAS Component	Total Conserved miRNA Targets	Total Poorly Conserved miRNA Targets	Overall Total miRNA Targets
AGTR1 (AT_1_R)	0	56	56
AGTR2 (AT_2_R)	2	96	98
ACE	0	26	26
ACE2	1	57	58
AGT	1	31	32
REN	0	15	15
ATP6AP2 (PRR)	4	49	53
MAS1 (MasR)	0	12	12
LNPEP (AT_4_R)	5	42	47

*Source: TargetScan (April, 2009). The number of potential targets is dramatically dependent upon the algorithm utilized.

**Table 2:** Patrocles* algorithm-predicted RAS component miRSNPs.

RAS Component	dbSNP#	Hetero	dbSNP	bp from Stop Codon	Loss of Function miRSNP	Gain of Function miRSNP
**AGTR1 (AT_1_R)**	rs5184	0.069	A>G	2	miR-668	
	rs56343250	N.D.		40	mR-1237, -1248	
	rs5185	0.130	T>G	70	miR-302c, -573	miR-143*, -301a
	rs12721277	0.028	G>A	82	miR-579	
	**rs5186**	**0.500**	**A>C**	**86**	**miR-155**	
	rs5187	N.D.	A>G	93	miR-562, -548n	miR-646
	rs1799870	0.055	C>T	135		
	rs5188	0.109	G>A	317		miR-1197
	rs55707609	N.D.	A>T	335	miR-299-3p	
	rs5189	0.500	G>T	437		miR-570
	rs12721276	0.061	C>A	461		miR-128, -27a
	rs12721275	0.055	C>T	484	miR-143*	
	rs12721274	0.028	T>C	556	miR-641	
	rs440881	N.D.	A>C	565	miR-30b	
	rs1051649	N.D.	C>T	680	miR-1197	miR-7
	rs35533650	N.D.	A>G	704		
	rs380400	0.500	A>G	798		
	rs35393661	N.D.	->AT	803		

**AGTR2 (AT_2_R)**	rs34589510	N.D.	->T	57		
	rs5193	0.241	G>T	199		
	rs5194	0.499	A>G	205		miR-1229
	rs11091046	0.496	A>C	501		
	rs17231436	0.131	G>C	581	miR-384	
	rs41312570	N.D.	C>T	645	miR-301a, -130a	
	rs17231443	0.025	C>G	816		miR-548f, -570
	rs17237806	0.050	C>T	837		
	rs12858432	N.D.	C>T	906		
	rs12845035	0.030	C>G	1103	miR-361-3p	
	rs17237820	0.073	A>T	1111	miR-548a-3p	
	rs17231478	0.038	G>T	1185		
	rs17237827	0.025	A>G	1274		
	rs17231450	0.050	C>A	1318	miR-150*	
	rs17231457	0.050	A>C	1536		miR-571

ACE	None					

ACE2	None					

AGT	rs61762526	0.007	G>T	33		
	rs5042	0.008	C>T	40	miR-486-3p	
	rs4753	0.021	G>C	158		miR-337-5p
	rs61751079	0.002	G>A	159		miR-639, -539
	rs5043	0.021	T>C	167		
	rs61751080	0.002	T>A	168		
	rs11684	N.D.	C>T	184	miR-103, -107	
	rs1803103	N.D.	G>T	187	miR-483-3p	
	rs15022	N.D.	G>T	188	miR-483-3p	
	rs1803104	N.D.	A>G	316		
	rs1803106	N.D.	A>C	338	miR-129-5p	miR-335*
	rs61751081	0.002	C>T	350		
	rs5044	0.029	T>G	423		miR-1283, -606
	rs61762525	0.004	C>T	473		miR-548l, -559
	rs7079	0.312	C>A	556	miR-218-1*, -584	
	rs55720804	0.035	C>-	573		
	rs61751082	0.023	C>-	575		

REN	rs11799601	0.500	C>A	48		miR-326, -330-5p
	r11571124	0.041	C>T	145	miR-138	miR-150*

ATP6AP2 (PRR)	rs5963816	0.296	A>T	266	miR-664	
	rs6609080	0.186	A>G	358	miR-1179	
	rs9062	N.D.	G>T	654	miR-508-5p	miR-410
	rs10536	0.334	A>G	761	miR-802	miR-140-3p, -497*
	rs1060063	0.340	T>C	809		

MAS1 (MasR)	None					

LNPEP (AT_4_R)	rs17087239	0.004	C>T	61		miR-992, -15b
	rs39602	0.488	C>G	217		
	rs75912980	0.180	T>A	364		miR-1225-5p, -9
	rs1057808	0.014	T>C	408		miR-922, -15b
	rs3756618	0.028	A>T	501	miR-22*, -26b*	miR-223
	rs35838718	N.D.	A>-	561		
	rs79818663	0.105	G>T	594		
	rs77639920	N.D.	C>T	614	miR-664*	
	rs62377081	N.D.	G>T	695	miR-302a*, -1264	miR-548l

*Source: www.patrocles.org/.

**Table 3:** Physiological ramifications of RAS miRSNPs.

RAS Component	Loss of Function miRSNP	Physiological Result	Gain of Function miRSNP	Physiological Result
AGTR1 (AT_1_R)	AT_1_R↑	Hypertension Cardiac/vascular remodeling Atherosclerosis	AT_1_R↓	Antihypertensive Antiremodeling Anti-atherosclerosis
ACE	ACE↑		ACE↓	
AGT	AGT↑		AGT↓	
REN	REN↑		REN↓	
ATP6AP2 (PRR)	PRR↑		PRR↓	
AGTR2 (AT_2_R)	AT_2_R↑	Antihypertensive Antiremodeling Antiatherosclerosis	AT_2_R↓	Hypertension Cardiac/vascular remodeling Atherosclerosis
ACE2	ACE2↑		ACE2↓	
MAS1 (MasR)	MasR↑		MasR↓	
LNPEP (AT_4_R)	AT_4_R↑		AT_4_R↓	
